# A high-resolution gene expression atlas links dedicated meristem genes to key architectural traits

**DOI:** 10.1101/gr.250878.119

**Published:** 2019-12

**Authors:** Steffen Knauer, Marie Javelle, Lin Li, Xianran Li, Xiaoli Ma, Kokulapalan Wimalanathan, Sunita Kumari, Robyn Johnston, Samuel Leiboff, Robert Meeley, Patrick S. Schnable, Doreen Ware, Carolyn Lawrence-Dill, Jianming Yu, Gary J. Muehlbauer, Michael J. Scanlon, Marja C.P. Timmermans

**Affiliations:** 1 Center for Plant Molecular Biology, University of Tuebingen, 72076 Tuebingen, Germany;; 2 Cold Spring Harbor Laboratory, Cold Spring Harbor, New York 11724, USA;; 3 Department of Agronomy and Plant Genetics, University of Minnesota, Saint Paul, Minnesota 55108, USA;; 4 Department of Agronomy, Iowa State University, Ames, Iowa 50011, USA;; 5 Interdepartmental Bioinformatics and Computational Biology Program, Iowa State University, Ames, Iowa 50011, USA;; 6 Plant Biology Section, School of Intergrated Plant Science, Cornell University, Ithaca, New York 14853, USA;; 7 DuPont Pioneer, Agricultural Biotechnology, Johnston, Iowa 50131, USA

## Abstract

The shoot apical meristem (SAM) orchestrates the balance between stem cell proliferation and organ initiation essential for postembryonic shoot growth. Meristems show a striking diversity in shape and size. How this morphological diversity relates to variation in plant architecture and the molecular circuitries driving it are unclear. By generating a high-resolution gene expression atlas of the vegetative maize shoot apex, we show here that distinct sets of genes govern the regulation and identity of stem cells in maize versus *Arabidopsis.* Cell identities in the maize SAM reflect the combinatorial activity of transcription factors (TFs) that drive the preferential, differential expression of individual members within gene families functioning in a plethora of cellular processes. Subfunctionalization thus emerges as a fundamental feature underlying cell identity. Moreover, we show that adult plant characters are, to a significant degree, regulated by gene circuitries acting in the SAM, with natural variation modulating agronomically important architectural traits enriched specifically near dynamically expressed SAM genes and the TFs that regulate them. Besides unique mechanisms of maize stem cell regulation, our atlas thus identifies key new targets for crop improvement.

The shoot apical meristem (SAM), positioned at the plant's growing shoot tip, harbors a population of pluripotent stem cells which serve as a persistent source of cells for postembryonic growth and organogenesis. A striking aspect of meristems is the tremendous diversity in morphology seen across plant species (Steeves and Sussex 1989). How this diversity relates to variation in overall plant architecture is unclear. SAM morphology does not seem to follow phylogeny (Steeves and Sussex 1989). This implies that the architectural diversity of the angiosperms is elaborated postmeristematically and that the main function of the SAM is to balance stem cell proliferation with organogenesis. Contrary to this concept, quantitative variation in SAM structure in maize is correlated with adult morphological traits such as node number and flowering time (Leiboff et al. 2015). This suggests that variations in adult plant architecture may be determined in part by regulatory circuits acting in the SAM and that such regulatory networks form targets for selection in the improvement of agronomically important traits.

The nature of these regulatory networks remains unclear. Much of our understanding of SAM function originates from studies in *Arabidopsis*. These illustrate that gene expression within the growing meristem is precisely coordinated in a highly spatial and temporal manner. Mobile signals, mechanical inputs, and environmental cues all provide positional information to specify cell fates within the dynamic stem cell niche (Besnard et al. 2011; Pfeiffer et al. 2017). These inputs in part converge onto a negative feedback loop involving *WUSCHEL* (*WUS*) and *CLAVATA* (*CLV*) signaling that maintains stem cell number in the central zone (CZ) at the SAM tip. Additionally, these inputs, through their effects on auxin polar transport and signaling, link proliferation in the meristem to organ initiation in the peripheral zone (PZ) (Besnard et al. 2011; Pfeiffer et al. 2017).

Many of the recognized regulators of meristem function predate the origin of the angiosperms (Plackett et al. 2015). Nonetheless, whereas the roles for meristem regulators such as *WUS* and *CLV1/3* appear conserved across eudicots, substantial diversification in these regulatory pathways between monocot and eudicot lineages has been noted (Nardmann and Werr 2007). This raises the question as to whether diversity in SAM morphology is reflected at the level of molecular circuitries. Here, we have generated a high-resolution gene expression atlas of the vegetative maize shoot apex to address this question and to dissect the contribution of regulatory circuits acting in the SAM to quantitative variation in adult morphological traits.

## Results

### Tissue-specific genes contributing to cell identity

**Figure 1.**
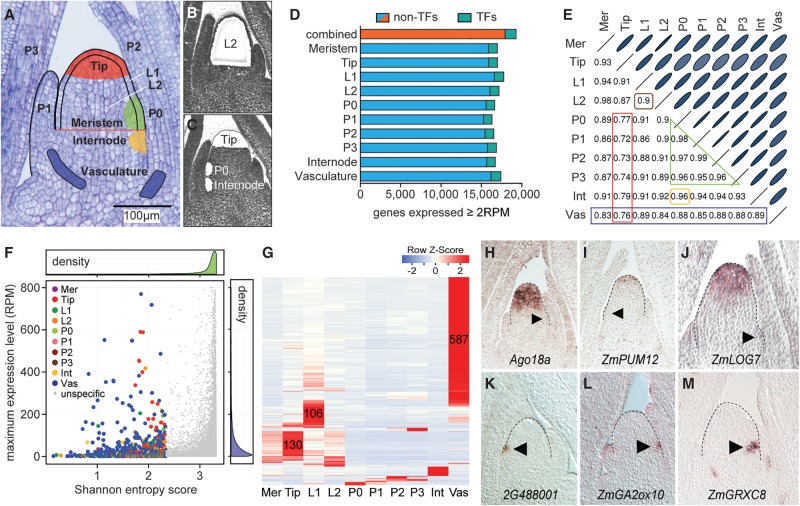
Tissue-specific genes contributing to cell identity. (*A*) Longitudinal section of a 14-d-old B73 seedling apex. The 10 domains/tissues captured are illustrated. (*B*,*C*) Sections after laser microdissection of the L2 (*B*), or Tip, P0, and internode (*C*). (*D*) The number of genes expressed ≥2 RPM varies only slightly across tissues. (*E*) Correlation analysis identifies the Tip as the most distinctive cell type (red), followed by the vasculature (blue). Overall expression in the two clonal layers is highly correlated (brown). Expression profiles of leaf primordia of successive plastochron (P) stages are also highly correlated (green), and closely match expression in the internode (yellow). Ellipse sizes inversely match correlation scores. (Mer) Meristem, (Int) internode, (Vas) vasculature. (*F*) Density plots show that most genes have high SE scores and low expression values. Genes with SE score <2.33 (colored) show tissue-specific expression. For visual simplification, only genes with expression values ≤ 800 RPM are shown. (*G*) Heat map of cell-type–specific genes shows most mark the vasculature, Tip, or L1. Primordium stage-specific transcripts were also identified. (*H*–*M*) In situ hybridization verifying specificity for select Tip- (*H*–*J*) and P0-specific genes (*K*–*M*). Meristem shape is outlined. Arrowheads, P0.

To identify gene expression signatures associated with meristem function in maize, we used laser microdissection to isolate cells from the following distinct structural and functional domains within the shoot apex of 14-d-old B73 seedlings: the entire meristem, the stem cell comprising meristem tip (hereafter referred to as Tip), the incipient leaf (P0) at the meristem periphery, the L1 and L2 lineage layers overlaying these meristem regions, developing leaf primordia at P1, P2, and P3, as well as the internode primordium and vasculature (Fig. 1A–C; Supplemental Fig. S1A,B). Collectively, transcripts of 19,278 genes, or about half of the 39,656 annotated maize genes, are detectable at levels ≥2 RPM in at least one of the 10 domains sampled (Supplemental Table S2). The number of genes expressed in discrete shoot apical domains varies little, and also their genome-wide expression profiles are highly correlated (Fig. 1D,E). This indicates that although the different domains within the shoot apex have highly distinct functional and anatomical characteristics, differential expression of a relatively small subset of genes underlies this specialization.

To identify such genes, we first used Shannon entropy (SE) (Schug et al. 2005) to define genes showing domain-specific patterns of expression. Of the 964 genes with a SE score <2.33 which, considering the partial overlap of the regions captured, were defined as domain-specific, most mark the vasculature (587), Tip (130), or L1 (106) (Fig. 1F,G; Supplemental Table S3). Genes such as *ZmLAX2*, *ZmRANBP2*, *brown midrib3*, *narrow sheath1*, *knotted1* (*kn1*), *aberrant phyllotaxy1*, *sparse inflorescence1*, and *barren stalk1* are among the domain-specific genes, as expected. This analysis, however, greatly increased the number of genes marking each tissue. Particularly, the large set of Tip-specific genes is important (Fig. 1F–J), as genes underlying maize stem cell identity have remained elusive. Additionally, genes specific to individual leaf stages, including the P0, had not been noted previously (Fig. 1K–M). These gene sets provide a powerful resource to infer tissue-specific enhancer elements, to modulate spatial patterns of gene expression, or to assign spatiotemporal origins to single-cell transcriptomic data (Denyer et al. 2019).

Genes with signaling-associated functions are overrepresented among the domain-specific genes, pointing to particular signaling pathways underlying cell fate decisions (Supplemental Table S4). For example, key cytokinin-synthesis genes show a Tip-specific pattern of expression, whereas gibberellic acid (GA)-associated genes are among the primordium-specific genes, and genes involved in auxin, abscisic acid, jasmonic acid and ethylene signaling predominate the vasculature (Supplemental Tables S3, S4). Such an organization of hormone activities will increase the possibilities for specific spatial interactions needed to coordinate the many cell fate decisions within the growing shoot. In addition, of the 48 genes encoding CLE signaling peptides (Goad et al. 2017), 17 are expressed in the apex, of which four show specificity for the vasculature, internode, or Tip (Supplemental Fig. S2A). The peptide derived from the single Tip-specific *CLE* gene is orthologous to rice *FCP1*. Although previously reported to be primordium-derived (Je et al. 2016), *ZmFCP1* is, in fact, specific to the Tip (Supplemental Fig. S2A,B) and could act through a *CLE*-*WOX* regulatory module within the SAM itself.

Besides genes connected to cell-cell signaling, transcripts for a substantial number of transcription factors (TFs) accumulate in a tissue- or domain-specific manner. Whereas 7% of all expressed genes encode TFs (Fig. 1D), consistent with a role in driving the primary molecular changes underlying cell identity, 117 of the 964 domain-specific genes represent TFs (>12%) (Supplemental Table S3). Expression of all 12 tissue-specific Dof TFs is limited to the vasculature, in line with their proposed role as master regulators of vascular development (Le Hir and Bellini 2013). However, for most TF families, expression is not obviously connected to a single cell or tissue type (Supplemental Table S3). Likewise, each tissue expresses a widely diverse set of TFs, pointing to combinatorial inputs from TFs on cell identity.

### Dynamic expression of individual gene family members defines cell identity

**Figure 2.**
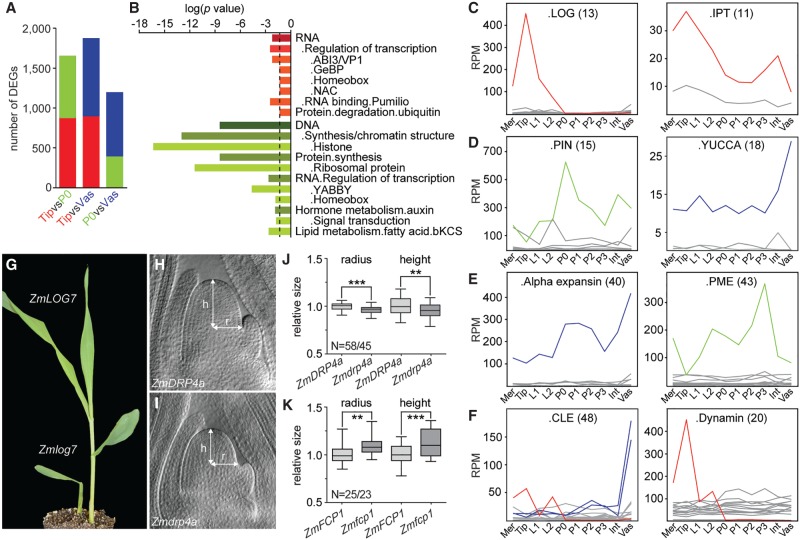
Differential expression of individual gene family members determines cell identity. (*A*) Pairwise differential gene expression analyses between Tip, P0, and vasculature show ∼10% of all expressed genes are differentially expressed (*q* < 0.01). Colors represent preferential expression within respective domains. (*B*) Enrichment analyses identified few functional categories overrepresented among Tip versus P0 DEGs. Dashed line, significance threshold (*P* = 0.05). (*C*–*F*) Individual genes within gene families show abundant and differential expression across the apex. Examples are shown for gene families functioning in cytokinin (*C*), auxin (*D*), cell wall (*E*), or other signaling processes (*F*). Number of family members is shown in parentheses. Only genes expressed ≥ 2 RPM are illustrated. Mer, meristem; Int, internode; Vas, vasculature. (*G*) *Zmlog7* mutants display meristem termination phenotypes. (*H*,*I*) Cleared shoot apices of wild type (*H*) and the small meristem mutant *Zmdrp4a* (*I*). (*J*,*K*) Box-and-whisker plots of SAM size measurements of *Zmdrp4a* (*J*) or *Zmfcp1* (*K*) and their respective wild-type siblings. (N) Number of wild-type/mutant apices measured. (**) *P* < 0.01, (***) *P* < 0.001, according to Student's *t*-test.

While SE identifies genes with a near on/off state in expression, more genes likely contribute in a quantitative manner to distinguish cell identities. We therefore next analyzed differentially expressed genes (DEGs) between the vasculature, Tip, and P0, the latter as a representative of the closely related leaf primordia and internode (Fig. 2A; Supplemental Table S5). Genes preferentially expressed in the vasculature largely overlap with the vascular-specific genes and show an overrepresentation for genes involved in signaling as well as cell wall homeostasis (Supplemental Table S6). In the pairwise comparison between the Tip and P0, which reveals transcriptional changes associated with the transition from stem cell to organ identity, genes preferentially expressed in the P0 are enriched for functions connected to actively dividing cells and auxin signaling (Fig. 2B; Supplemental Table S6), known features of primordium initiation. Additionally, consistent with TFs driving developmental programs, differences are seen for select TF families. For instance, the NAC, GeBP, and ABI3/VP1 families are overrepresented among Tip-enriched genes, whereas YABBY TFs, as shown previously, mark the P0 (Fig. 2B; Supplemental Fig. S1B; Juarez et al. 2004).

Perhaps the most striking feature stemming from this analysis, however, is the fact that few functional categories or pathways are enriched among the DEGs (Fig. 2B; Supplemental Table S6). This reflects an unexpected degree of subfunctionalization within gene families. DEGs from all three pairwise comparisons are annotated to function in numerous, widely diverse metabolic and cellular processes. Although these processes are typically represented by multigene families, single or highly select subsets of members within these families show a differential or tissue-specific pattern of expression (Fig. 2C–F). Besides the *CLE* example mentioned above, individual *LONELY GUY* (*LOG*), *ISOPENTENYL TRANSFERASE* (*IPT*), *PIN-FORMED* (*PIN*), and *YUCCA* genes involved in hormone metabolism/signaling are differentially expressed across these tissues (Fig. 2C,D; Supplemental Fig. S2C). Likewise, individual family members for cell wall modifying enzymes and redox regulation (e.g., expansins, pectin methylesterases, and glutaredoxins), known to act downstream from TFs and hormone signaling in meristem homeostasis and organogenesis (Schippers et al. 2016; Tognetti et al. 2017), are differentially expressed (Fig. 2E; Supplemental Fig. S2D,E). Key features of cell identity are thus regulated across tissues by a comparatively small but functionally highly diverse set of DEGs.

Moreover, the expression level of such DEGs across all 10 tissues sampled often far exceeds that of the remaining more uniformly expressed family members (Fig. 2C–F; Supplemental Fig. S2C–E). This predicts a more limited degree of redundancy. Indeed, putative loss-of-function alleles available for three DEGs with strong preferential expression in the Tip each show meristem phenotypes. Mutation of *ZmLOG7* conditions a meristem termination phenotype (Fig. 2G). This is contrary to *Arabidopsis* and rice, where *log* mutations have little or no effect on vegetative meristem size (Kurakawa et al. 2007; Tokunaga et al. 2012). In addition, mutations in the dynamin family member *ZmDRP4a* cause a reduction in SAM radius, whereas *Zmfcp1* mutants show a significant increase in SAM size (Fig. 2H–K; Je et al. 2016), consistent with the presence of a local *CLE-WOX* module regulating stem cell number.

Thus, the cellular mechanisms linking patterns of TF activity to the differentiation of distinct cell types are highly complex. Inputs from discrete signaling components converge onto combinatorially acting TFs, a subset of which is expressed in a tissue-specific manner, that drive the strong, differential expression of select, often individual, genes within gene families. These are predicted to function in a wide array of metabolic and cellular processes to confer distinctive properties onto functional domains within the apex.

### Molecular signatures underlying functional SAM domains

**Figure 3.**
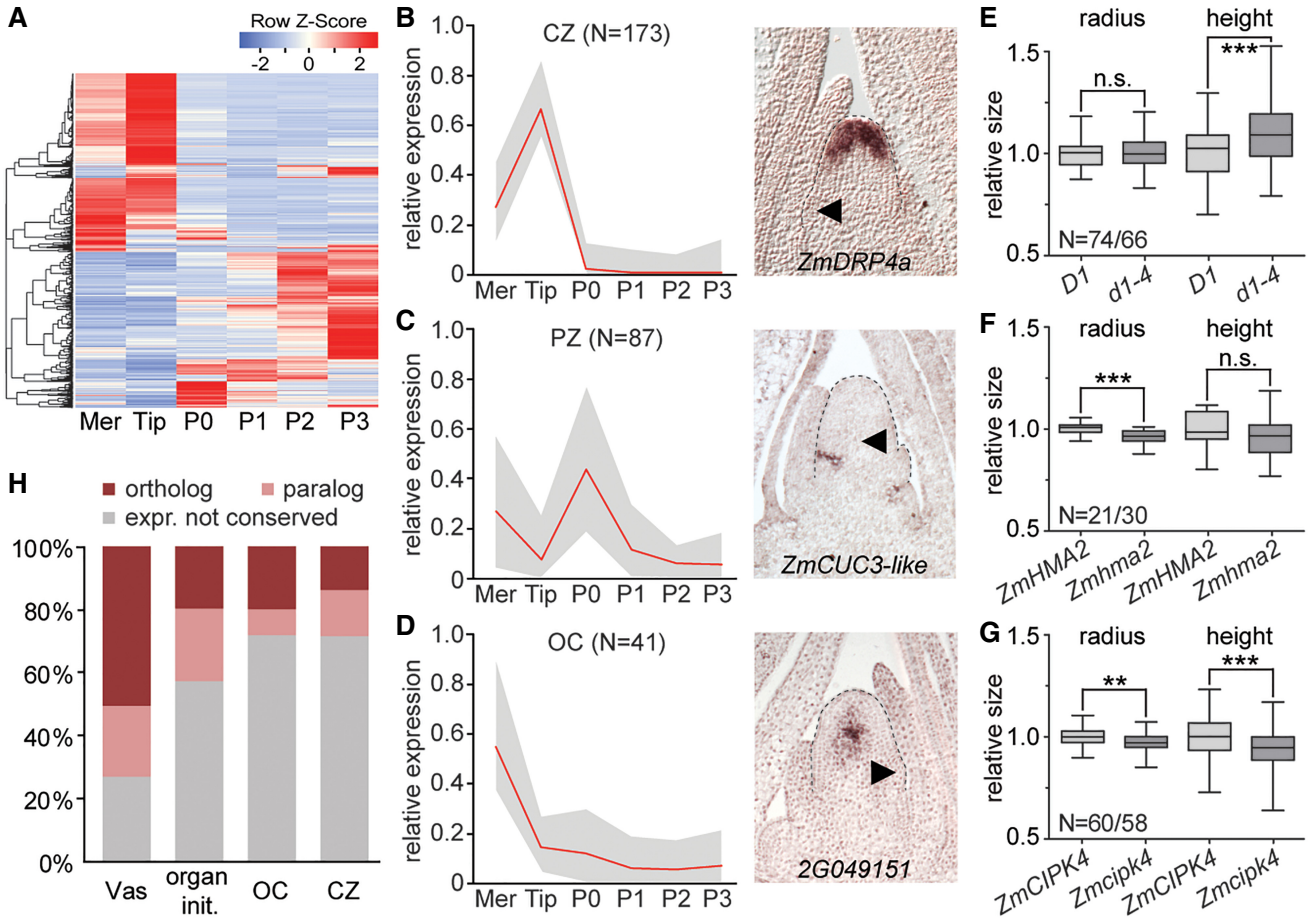
Divergent gene sets define functional SAM domains in maize versus *Arabidopsis*. (*A*) Expression heat map of genes dynamically expressed during the transition from stem cell (Tip) to P3 organ primordium. Genes of Cluster 1 whose expression changes minimally are excluded. (Mer) Meristem. (*B*–*D*) *Left*: composite expression profiles of gene clusters marking the CZ (*B*), PZ (*C*), or OC (*D*). *Right*: in situ hybridization patterns of select cluster members. Arrowheads, P0; N, number of genes in respective clusters; red line, mean expression; gray profile, range between highest and lowest values. (*E*–*G*) Mutations in the OC genes *D1* (*E*), *ZmHMA2* (*F*), and *ZmCIPK4* (*G*) affect meristem height, radius, or both. (N) Number of measured wild-type/mutant individuals. (n.s.) Not significant, (**) *P* < 0.01, (***) *P* < 0.001, according to Student's *t*-test. (*H*) Percentages of maize genes expressed specifically in vasculature, during organ initiation, or in the OC or CZ with a similarly expressed *Arabidopsis* ortholog (dark red) or related paralog (light red) (Yadav et al. 2014). Maize genes without an identifiable *Arabidopsis* ortholog or near paralog are not shown.

Besides the classically defined peripheral and central zones, studies in *Arabidopsis* revealed the presence of an organizing center (OC) positioned immediately below the CZ. The OC provides positional information required to specify stem cell fate and balances activities in the central and peripheral zones of the SAM (Pfeiffer et al. 2017). Cells in the OC are characterized by expression of the *WUS* TF (Mayer et al. 1998). However, whereas *WUS* orthologs in other eudicot species share this pattern of expression (Galli and Gallavotti 2016), *ZmWUS1* expression within the vegetative SAM is not conserved (Nardmann and Werr 2006). Likewise, the *CLV1* ortholog *thick tassel dwarf1* is expressed in leaf primordia (Nardmann and Werr 2007), and *ZmFCP1*, rather than the maize *CLV3* ortholog, specifically marks the SAM tip (Supplemental Fig. S2A,B). This indicates substantial diversification in the *WUS-CLV* signaling pathway between monocot and eudicot species and leaves open the identity of an OC in the morphologically distinct maize SAM. To address this question and to identify molecular signatures that distinguish the functional SAM domains, we clustered genes based on their transcript profiles across the meristem, Tip, P0, and P1–P3 leaf primordia (Fig. 3A). Inclusion of the latter enhanced the ability to identify SAM-specific signatures and allowed following the transition from indeterminate stem cell through differentiation.

Among the 46 clusters obtained, two large gene clusters show increased or decreased expression with leaf ontogeny (Supplemental Fig. S3A,B), providing numerous markers for comparative analyses of leaf development. Additionally, few clusters show expression profiles expected for meristem core genes, with high relative expression in both meristem and Tip and minimal expression during organogenesis (Supplemental Fig. S3C; Supplemental Table S7). These clusters point to the presence of regulatory circuits promoting general meristem identity that presumably interact with genes specific to the CZ, OC, and lineage layers to specify regional identities within the SAM.

Several clusters show expression profiles consistent with expectations for the functional domains of the SAM. Two CZ-clusters with 173 genes (Fig. 3B) comprise mostly Tip-specific genes but also genes expressed in both the Tip and vasculature, possibly reflecting a general stem cell function, as well as genes that specifically mark the L1 (e.g., *ZmWOX9b*, *ZmWOX9c*) or L2 (e.g., *ZmFCP1)*, predicting layer-specific contributions to the CZ (Supplemental Fig. S3D). In addition to P0-specific genes, the four PZ-clusters, with 87 genes total (Fig. 3C), include genes connected to leaf initiation (e.g., *ZmWOX3a, Arf3b, ZmGA2ox*, and *fused leaves1*), boundary formation (e.g., *ZmCUC3-like*), and axillary meristem formation (e.g., *barren inflorescence2*) (Supplemental Table S7). RNA in situ hybridization verified that selected genes in these clusters indeed show the predicted domain-specific patterns of expression (Figs. 1H–M, 3B,C).

Genes expressed at a position equivalent to that of the *Arabidopsis* OC are predicted to show high expression in the meristem overall and minimal expression in the Tip and leaf primordia. This profile is seen in three clusters, comprising 41 genes total (Fig. 3D). In situ hybridization shows that transcripts for a gene of unknown function representative of these clusters localize to a small group of cells at the SAM center below the CZ, recapitulating the canonical OC expression pattern of *WUS* (Fig. 3D; Mayer et al. 1998). Further, in line with a role as a signaling center critical for balancing stem cell maintenance and organogenesis, genes encoding the GA 3-oxidase *dwarf plant1* (*D1*), *ZmBAS1*, a LRR receptor kinase, as well as proteins related to calcium-, redox-, and sugar-based signaling, are included in these clusters (Supplemental Table S7). Moreover, three of the six genes with available loss-of-function alleles display quantitative effects on meristem height (*d1*), radius (*Zmhma2*), or both (*Zmcipk4*) (Fig. 3E–G).

Genes in these clusters thus identify a domain in the maize SAM equivalent in position and function to the *Arabidopsis* OC. Ten TFs, each representing a distinct family, are among the OC genes. However, neither *ZmWUS1* nor any other WOX member shows this expression signature. In maize, the activity of a central signaling center required to balance cell fates within the shoot stem cell niche can thus be separated from WOX activity, pointing to divergence in the molecular networks underlying meristem function in maize compared to dicot species.

### Divergent molecular circuitries underlie SAM function in maize and *Arabidopsis*

To examine the extent of such divergence and to assess the degree to which morphological diversity between the maize and *Arabidopsis* SAM is reflected at the level of molecular circuitry, we asked whether *Arabidopsis* homologs of the maize SAM domain-specific genes show analogous patterns of expression. To this end, we took advantage of two previously generated atlases for the *Arabidopsis* apex (Yadav et al. 2014; Tian et al. 2019), which yielded comparable results. For about one third of maize domain-specific genes, an *Arabidopsis* ortholog could not be identified. This percentage is unexpected given that the origin of the layered meristem predates the divergence of monocot and dicot lineages (Gifford 1954) and that merely 19% of all expressed maize genes lack an identifiable *Arabidopsis* ortholog (Supplemental Table S8). Moreover, whereas ∼73% of maize vasculature-specific genes have an *Arabidopsis* ortholog or close paralog with a vascular enriched pattern of expression, only 28% of remaining maize OC and CZ genes have a close *Arabidopsis* relative whose pattern of expression is conserved (Fig. 3H; Supplemental Fig. S3F; Supplemental Table S8). Also, genes marking the incipient primordium (organ initiation clusters [Supplemental Table S7]) show more extensive expression conservation (Fig. 3H; Supplemental Fig. S3F).

Aside from *D1,* the signaling-related genes connected to the maize OC lack homologs in *Arabidopsis* with a similar pattern of expression, and only two of the 10 TFs marking the maize OC have related genes enriched in the OC of *Arabidopsis* (Supplemental Table S8). Within the CZ, particularly genes involved in transcriptional and posttranscriptional regulation, such as TFs, *ARGONAUTE 5 (AGO5)*, and *PUMILIO* (*PUM*) genes, show a conserved pattern of expression (Supplemental Table S8). AGO and Pumilio proteins are also required for germline and stem cell maintenance in animals (Siomi and Kuramochi-Miyagawa 2009; Slaidina and Lehmann 2014), pointing to possible fundamental features of stem cell regulation. Thus, while select processes are shared, highly divergent sets of genes define the OC and CZ in maize and *Arabidopsis*. This not only indicates crucial differences in the regulation of stem cell homeostasis but also that the stem cell state itself is distinguished by varying activities in processes related to signaling, chromatin, redox state, and more that can be tuned via differential expression of a variety of genes. The finding that quantitative expression level changes in hundreds of genes involved in a wide array of metabolic and cellular functions distinguish the CZ from leaf primordia supports this notion (Fig. 2A; Supplemental Table S5).

### Complex TF signatures drive cell identities

**Figure 4.**
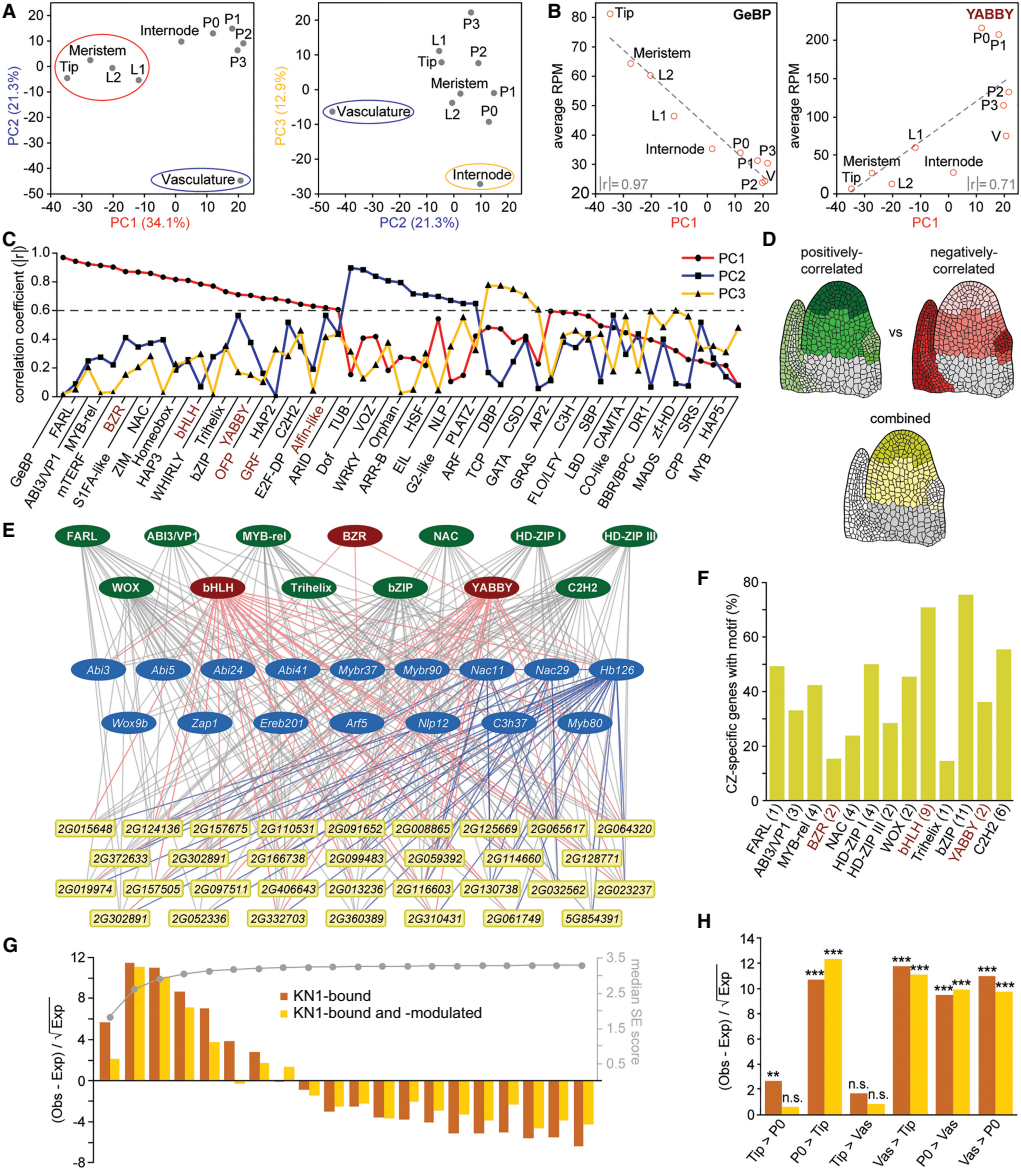
Combinatorial effects of multiple TFs distinguishes cell identities. (*A*) PCA showing TF expression across domains mirrors trends of genome-wide expression profiles with PC1 (red) and PC2 (blue) separating Tip and vasculature, respectively. PC3 (yellow) distinguishes the internode, and leaf primordia group together. (*B*) Mean TF family expression across domains correlates with given PCs. Examples are shown for TF families either positively (*left*) or negatively correlated (*right*) to PC1. Absolute correlation coefficients (│r│) are indicated. (V) Vasculature. (*C*) Overview of absolute correlation coefficients (│r│) for each TF family to each major PC. A threshold of 0.6 (dashed line) was used as a correlation cutoff. Mean expression of TF families shown in red is negatively correlated to meristem identity. (*D*) Diagrams illustrating that combined expression values for all TFs positively (green) and negatively correlated (red) to PC1 together can govern cell-type specificity (yellow). Gray, not examined. (*E*) Visualization of a GRN for select CZ-specific genes (yellow and blue) with combinatorial and hierarchical interactions from TF families positively (green) and negatively correlated (red) to PC1, as well as individual CZ-specific TFs (blue). (*F*) Percentage of CZ-specific genes with binding sites for PC1-correlated TF families in their promoter. The number of tested motifs is given in parentheses. TF families shown in red are negatively correlated to PC1. (*G*) Distribution of KN1-bound (dark orange) and KN1-bound and -modulated (light orange) targets among apex-expressed genes grouped into 20 bins based on SE score (*right y*-axis) shows KN1 primarily targets dynamically expressed genes. (Obs) Observed, (Exp) expected. (*H*) DEGs targeted by KN1 are preferentially expressed in organ primordia and vasculature. (n.s.) Not significant, (**) *P* < 0.01, (***) *P* < 0.001, based on χ^2^ test with Yates’ continuity correction.

The expression divergence between maize and *Arabidopsis* raises the question of how cell fates within the maize SAM are specified. Many TFs show tissue-specific or differential patterns of expression, predicting a causal relationship to the expression changes characterizing individual cell and tissue types. To address this, we performed a principal component analysis (PCA) considering expression values of all TFs across the 10 domains under study. This identified three principal components that distinguish meristematic tissues (PC1), vasculature (PC2), and internode (PC3), respectively (Fig. 4A; Supplemental Table S9). The spatial separation based merely on the expression profiles of TFs, which represent ∼7% of all expressed genes, thus mirrors the overall trends observed in correlation analysis (Fig. 1E), supporting the idea that defined sets of TFs underlie the expression changes that drive cell identity.

Further predicting causative relationships between specific TF families and tissue identity, the mean expression values of most TF families are strongly correlated with a given PC (Fig. 4B,C; Supplemental Table S9). Eleven of the 55 maize TF families (http://grassius.org/) are positively correlated to vascular identity (PC2) (Fig. 4C), including the earlier mentioned Dof TFs, type-B ARRs known to promote vascular identity (Yokoyama et al. 2007), as well as TUBBY and G2-like TFs, of which several show vascular-specific expression (Supplemental Table S3). The mean expression values for 23 TF families are highly correlated with PC1, of which 17 are positively correlated, showing highest expression in meristem tissues (Fig. 4B,C). Among them are the GeBP, FARL, NAC, and Homeobox (HB) TFs, which have a demonstrated link to meristem function (Vollbrecht et al. 2000; Aida and Tasaka 2006; Chevalier et al. 2008; Aguilar-Martínez et al. 2014). Mean expression for the remaining six families is negatively correlated with PC1, suggesting that they act as repressors of meristem identity and/or promote organogenesis. Indeed, YABBY, GRF, and OFP TFs either directly or indirectly repress KNOX gene function (Dai et al. 2007; Hay and Tsiantis 2010; Kuijt et al. 2014; Wang et al. 2016).

Of the TF families positively correlated to PC1, the GeBP, HB, ABI3/VP1, and NAC TF families are overrepresented among Tip enriched genes in DE and SE analyses (Fig. 2B; Supplemental Table S4). In addition, 81 out of 583 PC1-correlated TFs are included in the meristem core or subdomain clusters (Supplemental Table S7). However, most TFs are expressed more broadly, and correlation to PC1 reflects the additive effect of more subtle quantitative expression differences from multiple TF family members across the vegetative apex. When considering the collective quantitative expression differences of all PC1-correlated TFs, this offers a basis for generating meristem- and CZ-specific patterns of expression (Fig. 4D). Positive PC1-correlated TFs show the highest cumulative expression in the meristem and the CZ particularly, whereas expression of TFs negatively correlated to PC1 is lowest in these tissues. When combined, the opposing effects of these TFs could conceivably bring about tissue specificity. Accordingly, the spatially restricted expression of meristem- and CZ-specific genes is predicted to reflect the combinatorial activities of multiple meristem-promoting and -repressing TFs.

### Combinatorial effects of diverse TFs promote stem cell fate

To test this hypothesis and to assess a contribution of PC1-correlated TFs to cell fate specification, we modeled a gene regulatory network (GRN) based on the occurrences of TF binding motifs within the promoters of CZ-specific genes. Indicative of functional regulatory interactions in vivo (Reece-Hoyes et al. 2013; Sparks et al. 2016), *cis*-regulatory elements for all 13 PC1-correlated TF families with available binding position weight matrices are highly enriched in proximal promoters of CZ genes relative to whole-genome incidence (Supplemental Table S10).

The GRN reveals a highly interconnected arrangement of possible transcriptional regulatory interactions. Each of the 13 PC1-correlated TF families can target a substantial number of CZ-specific genes (Fig. 4E,F; Supplemental Fig. S4A). For instance, both FARL and HD-ZIPI DNA-binding motifs are present in the promoters of nearly half the CZ-specific genes (Fig. 4F). Both TF families mediate transcriptional responses downstream from light signaling (Harris et al. 2011; Siddiqui et al. 2016), pointing to mechanisms allowing for plasticity in the specification of functional SAM domains in response to environmental cues. Conversely, the promoters of CZ-specific genes contain *cis*-regulatory motifs for, on average, five distinct PC1-correlated TF families, with some promoters containing binding sites for as many as 10 of the 13 families analyzed (Supplemental Table S11). This reinforces the idea that domain- or tissue-specific expression reflects the combinatorial actions of multiple TFs. Further, the combination of TF target sites in individual promoters varies considerably (Supplemental Table S11), suggesting that tissue specificity can come about in many ways.

Most promoters include binding sites for both TFs positively and negatively correlated to PC1 (Fig. 4E,F; Supplemental Fig. S4A), consistent with the idea that the spatially restricted expression of CZ genes reflects the combinatorial activities of both meristem-promoting and -repressing TFs. The GRN further shows hierarchical transcriptional regulation, with more broadly expressed PC1-correlated TFs converging on the promoters of CZ-specific TFs (Fig. 4E). Binding sites for both more broadly expressed and tissue-specific TFs are present in promoters of other CZ genes, generating a network configuration that would reinforce tissue specificity of cell fate determinants (Barolo and Posakony 2002; Niwa 2018). Given the overrepresentation of TFs among tissue-specific genes, such regulatory relationships appear a general feature underlying cell identities.

### KN1 promotes meristem fate by repressing organogenesis and differentiation

The above GRN points to substantial redundancy among TFs in meristem regulation. Nonetheless, mutations in *kn1*, which is part of the meristem core cluster (Supplemental Fig. S3C), can show a highly penetrant meristem termination phenotype (Vollbrecht et al. 2000). KN1 functions in generating differential patterns of expression across the apex, as its targets (Bolduc et al. 2012) are specifically enriched among dynamically expressed genes (Fig. 4G,H). KN1 particularly targets genes preferentially expressed in the vasculature and P0, where it is not itself expressed, and thus seems to regulate meristem activity primarily by repressing differentiation rather than promoting meristem identity. Supporting this notion, genes predicted to drive organogenesis, e.g., auxin signaling, cytokinin turnover, and cell wall remodeling, are overrepresented among KN1 targets (Supplemental Fig. S4B; Supplemental Table S12). Moreover, consistent with being a central hub in the CZ GRN, KN1 binds a substantial number (∼23%) of PC1-correlated TFs. However, only TFs negatively correlated to PC1 are enriched among the KN1 targets, whereas those positively correlated are depleted (Supplemental Table S13). Thus, KN1 is a master regulator of meristem activity that mediates indeterminacy by selectively targeting key transcriptional regulators and signaling pathways that promote organogenesis and differentiation.

### Dynamically expressed meristem genes modulate important architectural traits

**Figure 5.**
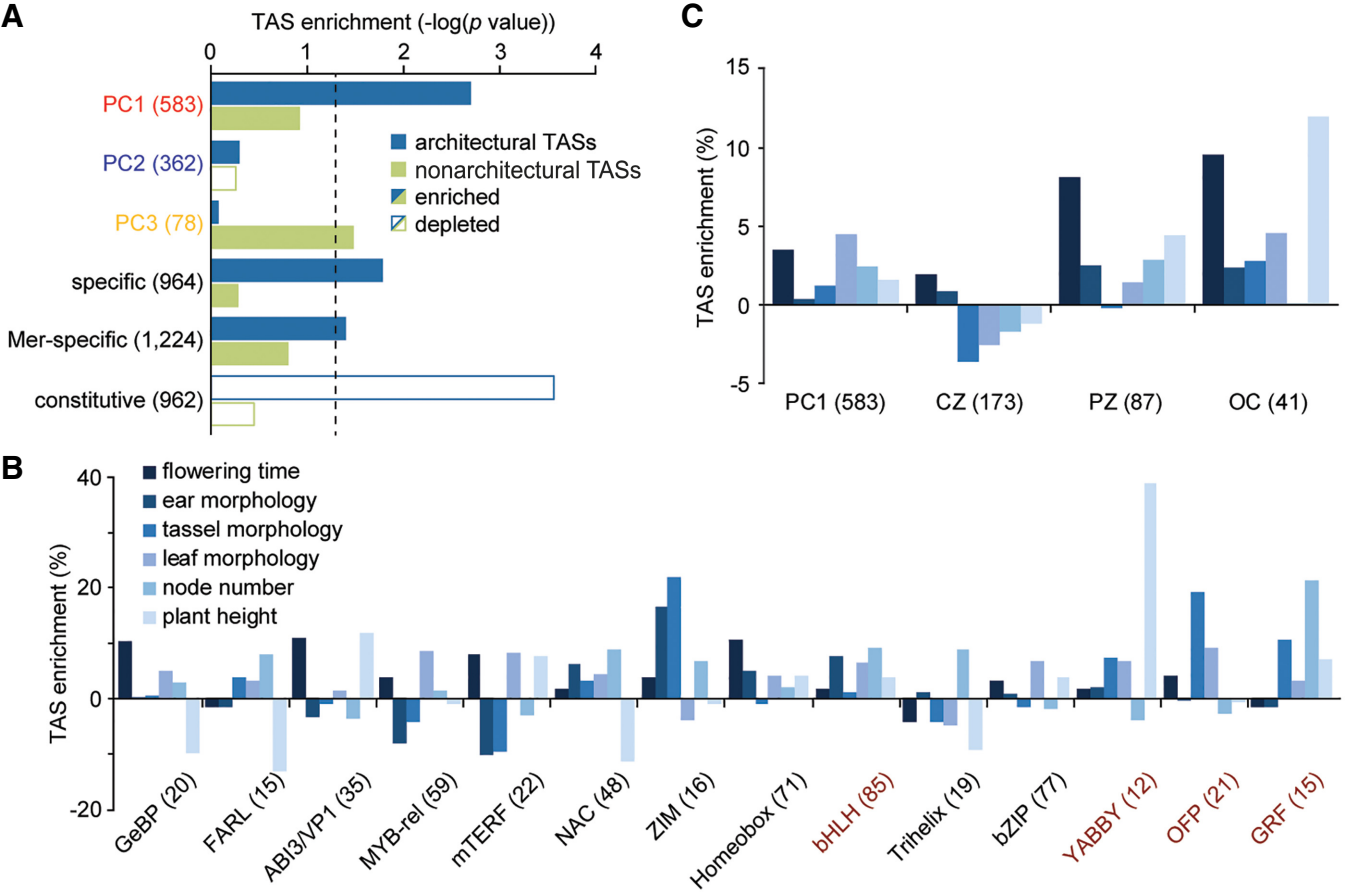
Dynamically expressed meristem genes modulate architectural traits. (*A*) Significance plot illustrating that natural variation underlying plant architectural traits is significantly enriched specifically near PC1-correlated TFs, dynamically expressed genes, and meristem-specific genes. In contrast, architectural TASs are strongly depleted near constitutively expressed genes. Dashed line, significance threshold (*P* = 0.05) based on χ^2^ test with Yates’ continuity correction. Input gene numbers are given in parentheses. (Mer) Meristem. (*B*) Enrichments of TASs near individual TF families either positively (black) or negatively correlated (red) to PC1 shows different TF families shaping distinct morphological features. (*C*) Enrichments of TASs influencing individual architectural traits near PC1-correlated TFs and genes marking the PZ and OC. TASs for most architectural traits are depleted near CZ-specific genes.

A combinatorial quantitative contribution from diverse TFs to cell fate specification confounds mutational analyses. We therefore took advantage of data from genome-wide association studies (GWAS) to address function and tested whether potential natural variation present at these loci contributes to the quantitative variation in plant morphology present among maize varieties. Individual traits measured in more than a dozen GWAS studies (see Supplemental Table S14 for details) were broadly classified as being architectural or nonarchitectural in nature. Collectively, ∼60% of SNPs associated with traits in either broad category are located within 10 kb of apex-expressed genes (Supplemental Table S14). Despite the stringent criteria applied (see Methods), PC1-correlated TFs are significantly enriched among expressed genes associated with plant architectural traits (Fig. 5A). In contrast, natural variation linked to disease and metabolic traits maps preferentially near TFs underlying PC3. A breakdown into individual architectural traits shows that different PC1-correlated TF families control distinct morphological features (Fig. 5B). For instance, natural variation near ABI3/VP1 and YABBY TFs is linked to diversity in plant height, whereas allelic diversity at Myb-rel and OFP TFs is strongly associated with leaf morphology traits and, at Trihelix and GRF TFs, with node number.

Plant morphological diversity is thus determined in part by TFs connected to cell fate decisions in the SAM. In addition, genes identified by SE to have a tissue-specific or dynamic pattern of expression are uniquely enriched near architectural Trait-Associated SNPs (TASs), whereas constitutively expressed genes are strongly depleted (Fig. 5A; Supplemental Fig. S5). This highlights the importance of polymorphisms near dynamically expressed genes in shaping morphological variation and identifies such genes as key targets during breeding selection. Among the dynamically expressed genes, members of the meristem core and subdomain clusters are significantly enriched again only near architectural TASs, affecting a variety of morphology traits (Fig. 5A,C; Supplemental Table S7). However, the subset of genes marking the CZ is depleted near SNPs underlying variation in the majority of adult plant characters. Instead, natural variation near genes marking cells of the OC or PZ strongly influences the overall morphology of the plant (Fig. 5C). Adult plant characters, including key agronomic traits, such as leaf angle, leaf shape, plant height, flowering time, and inflorescence morphology, are thus to a substantial degree associated with gene circuitries acting in the SAM.

## Discussion

The SAMs of most angiosperms share a basic functional organization. Meristems are nonetheless characterized by striking diversity in shape and size (Steeves and Sussex 1989), as nicely exemplified by the highly divergent morphologies of the maize and *Arabidopsis* SAM. Our study shows that this morphometric diversity is reflected at the level of molecular circuitry. The high-resolution gene expression atlas identified molecular signatures defining critical domains of the maize vegetative apex. This reveals that distinct sets of genes underlie the regulation and identity of stem cells in maize versus *Arabidopsis*. Nevertheless, expression of *ZmFCP1* specifically in the CZ of the maize SAM predicts the presence of a locally restricted *CLE-WOX* module to balance stem cell number and stably anchor the CZ to the growing shoot tip. In *Arabidopsis*, two opposing signaling centers provide relevant positional cues; mobile WUS from the OC promotes stem cell identity in distal cells, and epidermal-derived miR394 anchors the CZ to the SAM tip (Yadav et al. 2011; Knauer et al. 2013; Daum et al. 2014). In maize, the OC is not defined by WOX expression, suggesting an alternative mechanism to maintain a region of stem cell competence at the tip. An intriguing hypothesis is that, in the vegetative SAM of maize, the sources of these signals are displaced. Likely candidates to promote stem cell identity are *ZmWOX9b* and *ZmWOX9c*, which are both expressed in the L1 of the CZ. Consistent with this idea, *ZmFCP1* is primarily expressed in subepidermal layers, where it overlaps with its receptors FEA2 (Je et al. 2018) and FEA3 (Je et al. 2016). WOX9 protein could represent a steady but inward-directed stem cell-promoting factor that, in conjugation with hormones and other signals originating from the OC, provide the positional information required to stabilize stem cell activity in the growing niche.

The cellular mechanisms linking positional inputs to patterns of TF activity and the differentiation of distinct cell and tissue types are highly complex. Cell identities are distinguished by the differential expression of hundreds of genes involved in a wide array of metabolic and cellular processes. A surprising number of these DEGs are expressed at levels far exceeding those of other family members. Thus, reminiscent of divergent paralogs driving morphological innovation (Panchy et al. 2016), subfunctionalization of gene family members is a key feature underlying the differentiation of distinct cell types. Our findings further identify such single DEGs as prime targets via which to shape plant morphology and manipulate developmental traits critical to crop improvement.

The CZ GRN further predicts that genes affecting cell identity are targeted by both activating and repressing TFs and that more broadly expressed TFs act in a combinatorial and hierarchical manner with cell-type–specific TFs to define their spatially restricted patterns of expression. This point is substantiated by PC analysis, which in turn is supported by strong enrichment of architectural TASs specifically near PC1-correlated TFs. Network configurations in which target gene expression reflects the combinatorial additive and opposing effects of more general and locally restricted TFs is emerging as a general feature underlying developmental patterning (Barolo and Posakony 2002; Sparks et al. 2016; Reiter et al. 2017; Niwa 2018). These complex network architectures buffer gene expression by reducing the impact of mutations in individual TFs and, moreover, allow cells to discriminate true signaling inputs from background gene expression fluctuations to provide robustness (Sokolik et al. 2015). These features are particularly relevant to plants, given their sessile nature and the need to maintain stable developmental programs under highly variable conditions.

Nevertheless, this mechanism of robustness remains accompanied by vulnerabilities, as perturbations in highly connected nodes such as TFs at the top of a transcription cascade, can lead to a collapse of the entire network (Barabási and Oltvai 2004). For instance, misregulation of KN1, which targets nearly half the TFs negatively correlated with meristem fate, leads to severe developmental defects (Smith et al. 1992; Vollbrecht et al. 2000). However, expression of very few targets is altered in such mutants (Bolduc et al. 2012). Perhaps, KN1 functions as a pioneer factor that facilitates binding of other TFs to regulate target gene expression in this context-dependent manner (Reiter et al. 2017). However, given that KN1 primarily targets genes that function in organ primordia, an alternative, nonmutually exclusive view for the contribution of KN1 to SAM function is that it generates a state of default repression (Barolo and Posakony 2002). In this scenario, KN1 safeguards cells in the meristem from erroneously activating the differentiation program.

Although morphological diversity between plant species is thought to elaborate primarily postmeristematically (Steeves and Sussex 1989), our data show that maize plant architecture is to a significant degree regulated by molecular circuitries acting in the vegetative SAM. Both the high degree of TF connectivity and the broad spectrum of cellular processes underlying cell identity would allow a degree of circuitry evolvability. This is measured as quantitative variation, specifically in morphological traits. Our findings highlight a distinctive contribution from allelic diversity near dynamically expressed genes to phenotypic variation. In particular, polymorphisms near PC1-correlated TFs connected to cell fate decisions in the SAM, as well as genes expressed in the organogenic PZ or in the OC, which orchestrates the balance between stem cell maintenance and organogenesis, are associated with morphological diversity. Besides defining genes governing the identity and function of critical domains within the maize SAM, our gene expression atlas thus identifies key targets for selection in the improvement of agronomically important traits.

## Methods

### Plant materials

All analyses were performed on 14-d-old B73 seedlings grown under 16 h 24°C light and 8 h 20°C dark cycles. Mutant alleles for *ZmLOG7* (mu1030680, mu1052820), *dwarf plant1* (*d1-4*), *ZmCIPK4* (mu1046464), *ZmHMA2* (mu1076495), *GRMZM2G050234* (mu1037811, nonphenotypic), *GRMZM2G416817* (mu1070579, nonphenotypic), and *GRMZM2G168807* (mu1038844, nonphenotypic) were obtained from the Maize Genetics Cooperation Stock Center. DuPont Pioneer kindly screened for exon insertion alleles for *ZmFCP1* and *ZmDRP4a*. Transposon insertion alleles were introgressed for 3–4 generations into B73 (*Zmfcp1*) or T43 (all other mutations) prior to genetic and phenotypic analysis. See Supplemental Table S15 for gene IDs.

### Laser microdissection and RNA-seq library construction

Hand-dissected apices of 14-d-old B73 seedlings were fixed in acetone, embedded into paraffin, and sections of 8 µm spread on 1.0 PEN membrane slides (Zeiss), as described (Scanlon et al. 2009). After deparaffinization in xylene, cells of interest were captured into AdhesiveCap 500 tubes (Zeiss) using the PALM Micro-Beam system. To minimize variation, tissue samples were captured from sections from at least 10 individual apices for each of two biological replicates. RNA was extracted using the PicoPure RNA isolation kit (Arcturus), treated with DNase I (Qiagen), and amplified with the TargetAmp 2-Round aRNA Amplification kit 2.0 (Epicentre Biotechnologies). Single-end RNA-seq libraries were constructed using standard Illumina protocols (Illumina) and sequenced (100 bp) on the Illumina HiSeq 2000 platform.

### Gene expression analyses

The nucleotides of each raw read were scanned for low-quality bases. Bases with a Phred quality value <15 (out of 40), i.e., with error rates ≤3%, were removed by Data2Bio's trimming pipeline. Trimmed reads were aligned to the B73 RefGen_V3 using GSNAP (Wu and Nacu 2010), and uniquely mapped reads allowing ≤2 mismatches every 36 bp and less than 5 bases for every 75 bp as tails were used for subsequent analyses. Read counts per gene were computed using B73 gene annotation version FGSv5b. Library metrics are listed in Supplemental Table S1. Gene expression levels were normalized to RPM rather than RPKM, as linearly amplified RNA captures the 3′ 400–500 nucleotides of transcripts (Scanlon et al. 2009). Relatedness across tissue samples was determined based on the expression values of all genes expressed in each pairwise comparison using Pearson's correlation in R (R Core Team 2015). Subsequent analyses were performed on genes with a mean expression value ≥2 RPM in at least one of the 10 tissues sampled. Differential gene expression was determined using the DESeq (Anders and Huber 2010) package in R (R Core Team 2015) with default parameters and a BH-corrected *P*-value <0.01 cutoff (Benjamini and Hochberg 1995). Cell-type–specific genes were identified by Shannon entropy (Schug et al. 2005). The SE density distribution for all expressed genes fits a χ^2^ distribution (*P* < 2.2 × 10^−16^, Pearson's χ^2^normality test). SE scores <2.33 were considered as domain-specific to account for overlaps among some of the domains analyzed. For cluster analysis, genes showing a greater than or equal to twofold expression change in any two-way comparison between meristem, Tip, P0–P3 were identified using the Cluster Affinity Search Technique. Cluster analysis on the differentially expressed genes was conducted in the MultiExperiment Viewer (MeV) software package using Spearman's rank correlation as a distance metric and a threshold parameter of 0.8 according to Ben-Dor and Yakhini (1999). Heat maps were generated in R (R Core Team 2015) using the heatmap.2 function in the gplots package (https://rdrr.io/cran/gplots/).

### Enrichment analysis

MapMan annotations of the maize filtered gene set (v5b.60 [Usadel et al. 2009]) were used for functional enrichment analyses using hypergeometric distribution-based enrichment testing with GOseq (Young et al. 2010). TFs and hormone-related genes were manually annotated based on information from Grassius (http://grassius.org) (Yilmaz et al. 2009) and published work, or through identification of maize homologs of known *Arabidopsis* genes using the paralog search tool in BioMart (http://www.gramene.org) (Tello-Ruiz et al. 2018). Enrichments were determined using Fisher's exact test and reported as Benjamini-Hochberg–adjusted *P*-values (Benjamini and Hochberg 1995). Pathway analyses were performed with the ClueGO plug-in in Cytoscape (Shannon et al. 2003; Bindea et al. 2009) with standard settings on the GO/MolecularFunction, GO/BiologicalProcess, and KEGG databases.

### PCA and TF correlation analyses

An expression matrix of all expressed TFs was compiled and, after standardization, PCA was conducted using the Prcomp function in R (R Core Team 2015). Recognizing that TFs belonging to the same family are not always functionally interchangeable but that similarity within TF families is far greater than between families, the contributions of TF families to each principal component was estimated based on the correlations between principal component variables and the average expression values for each TF family using the Corrgram package in R (R Core Team 2015).

### *cis*-regulatory motif enrichment

To determine enrichment of *cis*-regulatory elements for PC1-correlated TFs within the promoters of Tip-specific genes, we used the computational prediction pipeline described in Eveland et al. (2014) that leverages the Search Tool for Occurrences of Regulatory Motifs (STORM) from the Comprehensive Regulatory Element Analysis and Detection (CREAD) suite of tools (Smith et al. 2006). Enrichment scores for 51 distinct position weight matrices (PWMs) for 13 PC1-correlated TF families obtained from Franco-Zorrilla et al. (2014) and Mathelier et al. (2016) were calculated based on their occurrence within promoter regions spanning 1 kb upstream of to 500 bp downstream from the transcription start site in CZ-specific genes over the complete B73 filtered gene set (v5b.60) (Supplemental Table S10). The Gene Regulatory Network was constructed in Cytoscape (Shannon et al. 2003).

### KN1 target enrichment

Bound, and bound and modulated KN1 targets were obtained from Bolduc et al. (2012), of which 2960 and 574 are expressed within the apex, respectively. Target enrichments were calculated as divergence from expectation using the following formula, (Observed−Expected)/√(Expected). Significance was calculated using a χ^2^ test with Yates’ continuity correction in R (R Core Team 2015).

### Trait-associated SNP enrichment

Genes located within 10 kb of SNPs associated with architectural traits and nonarchitectural traits were identified in R (Supplemental Code; R Core Team 2015). See Supplemental Table S14 for a complete list of trait-associated SNPs and their classifications. Enrichments over the occurrence of all expressed genes near trait associated SNPs were calculated as divergence from expectation. Stringency was applied by removing possible redundancies among trait associations by (1) considering genes containing multiple SNPs associated with a given trait only once, and (2) counting genes associated with either multiple architectural, or respectively multiple nonarchitectural traits, only once. Significance was calculated using a χ^2^ test with Yates’ continuity correction in R (R Core Team 2015).

### RT-PCR and in situ hybridization

For RT-PCR, 4 µg RQ1 DNase (Promega)-treated amplified RNA was converted into cDNA using the SuperScript III First-Strand Synthesis System (Invitrogen) with random hexamer primers according to the manufacturer's protocol. In situ hybridizations were performed on apices of 14-d-old B73 seedlings according to Javelle and Timmermans (2012). Gene-specific primers used in these analyses are listed in Supplemental Table S15.

### Meristem size measurements

Hand-dissected apices of mutant and nonmutant siblings were vacuum infiltrated for 2× 15 min in FAA (10% formaldehyde, 5% acetic acid, 45% ethanol solution) and fixed overnight in fresh FAA on a shaker at 4°C. Dehydration was performed on a shaker at 4°C for 1 h in 70%, 85%, 95%, and 100% ethanol, respectively, followed by 1 h in 100% ethanol at room temperature (RT). Apices were cleared overnight at RT on a shaker with ethanol:methyl salicylate (1:1), followed by 1 d in 100% methyl salicylate at RT, changing the solution once. Images were acquired with Nomarski optics on a Leica DMRB transmitted light microscope connected to a MicroPublisher 5.0 RTV camera (QImaging). Meristem height and radius were measured at the height of the P1 cleft from near-median longitudinal optical sections. Values were determined from two independently introgressed lines and normalized to the height and radius of wild-type siblings. Significance was determined by an unpaired two-tailed Student's *t*-test in GraphPad Prism.

### *Arabidopsis* expression analysis

*Arabidopsis* orthologs and paralogs of domain-specific maize genes were identified using BioMart v.07 Plant Genes 56 (http://www.gramene.org) (Tello-Ruiz et al. 2018). Expression profiles for *Arabidopsis* domains AtHB8 (xylem), S17shoot (phloem), WUS (OC), CLV3 (CZ), FIL (organ primordia), HDG4 (meristematic L2), HMG (meristematic L1), KAN1 (outer PZ), and LAS (organ boundary) were obtained from Yadav et al. (2014). To accommodate the partial overlap between domains, the following comparatively lax criteria were applied to determine tissue specificity: vasculature, relative AtHB8 or S17shoot expression >25% and expression in all other domains <20%; organ initiation, exclude S17shoot and AtHB8 expression, relative FIL or LAS expression >25%, expression in KAN, CLV3, and WUS <20%; OC, exclude S17shoot and AtHB8 expression, relative WUS expression >25%, expression in FIL, KAN, LAS, HMG, and HGD4 <20%; CZ, exclude S17shoot and AtHB8 expression, relative CLV3 expression > 25%, expression in FIL, KAN, LAS <20% (see Supplemental Fig. S3E; Supplemental Table S8). For a second independent analysis (Supplemental Fig. S3F), *Arabidopsis* orthologs and near paralogs of domain-specific maize genes were cross-referenced to genes identified as enriched (FC > 2) during organ initiation (FIL and LAS domains), in the OC (WUS domain), or in the CZ (CVL3 domain) by Tian et al. (2019) (Supplemental Table S8).

## Data access

All raw and processed sequencing data generated in this study have been submitted to the NCBI Gene Expression Omnibus (GEO; https://www.ncbi.nlm.nih.gov/geo/) under accession number GSE137715 and to the NCBI Sequence Read Archive (SRA; https://www.ncbi.nlm.nih.gov/sra) under accession number SRP101301.

## Supplementary Material

Supplemental Material
