# Supplementary material for: A high-resolution gene expression atlas links dedicated meristem genes to key architectural traits
Source: Genome Res. 2019 Dec;29(12):1962–73. doi: 10.1101/gr.250878.119 (PMC6886502; doi:10.1101/gr.250878.119)
Supplement: Supplemental Material [file supp_29_12_1962__index.html]

A high-resolution gene expression atlas links dedicated meristem genes to key architectural traits — A high-resolution gene expression atlas links dedicated meristem genes to key architectural traits — Supplemental Material 

# A high-resolution gene expression atlas links dedicated meristem genes to key architectural traits

## Supplemental Material

- Supplemental\_Figures.pdf
- Supplemental\_Tables.xlsx
- Supplemental\_Code.zip
