# Supplementary material for: A high-resolution gene expression atlas links dedicated meristem genes to key architectural traits
Source: Genome Res. 2019 Dec;29(12):1962–73. doi: 10.1101/gr.250878.119 (PMC6886502; doi:10.1101/gr.250878.119)
Supplement: Supplemental Material [file supp_gr.250878.119_Supplemental_Figures.pdf]

A high-resolution gene expression atlas links dedicated meristem genes to key architectural traits

Steffen Knauer[1,2,10], Marie Javelle[2,8,10] , Lin Li[3], Xianran Li[4], Xiaoli Ma[1], Kokulapalan Wimalanathan[5,9,], Sunita Kumari[2], Robyn Johnston[6], Samuel Leiboff[6], Robert Meeley[7], Patrick S. Schnable[4], Doreen Ware[2], Carolyn Lawrence-Dill[4,5], Jianming Yu[4], Gary J. Muehlbauer[3], Michael J. Scanlon[6] and Marja C.P. Timmermans[1,2]*

[1] Center for Plant Molecular Biology, University of Tuebingen, 72076 Tuebingen, Germany

[2] Cold Spring Harbor Laboratory, Cold Spring Harbor, NY 11724, USA

[3] Department of Agronomy and Plant Genetics, University of Minnesota, Saint Paul, MN 55108, USA

[4] Department of Agronomy, Iowa State University, Ames, IA 50011, USA

[5] Interdepartmental Bioinformatics and Computational Biology Program, Iowa State University, Ames, IA 50011, USA

[6] Plant Biology Section, School of Intergrated Plant Science, Cornell University, Ithaca, NY 14853, USA

[7] DuPont Pioneer, Agricultural Biotechnology, Johnston, Iowa 50131, USA

[8] Present address: Biogemma, CRC, route d'Ennezat, 63720 Chappes, France

[9] Present address: SOPHiA Genetics, Boston, MA 02116, USA

[10] These Authors contributed equally to this work

* Author of Correspondence: Marja CP Timmermans

Email: marja.timmermans@zmbp.uni-tuebingen.de

Tel.: ++49 7071 29 78099

Table of Contents

Supplemental Figure 1

Supplemental Figure 2

Supplemental Figure 3

Supplemental Figure 4

Supplemental Figure 5

Supplemental Figure 1

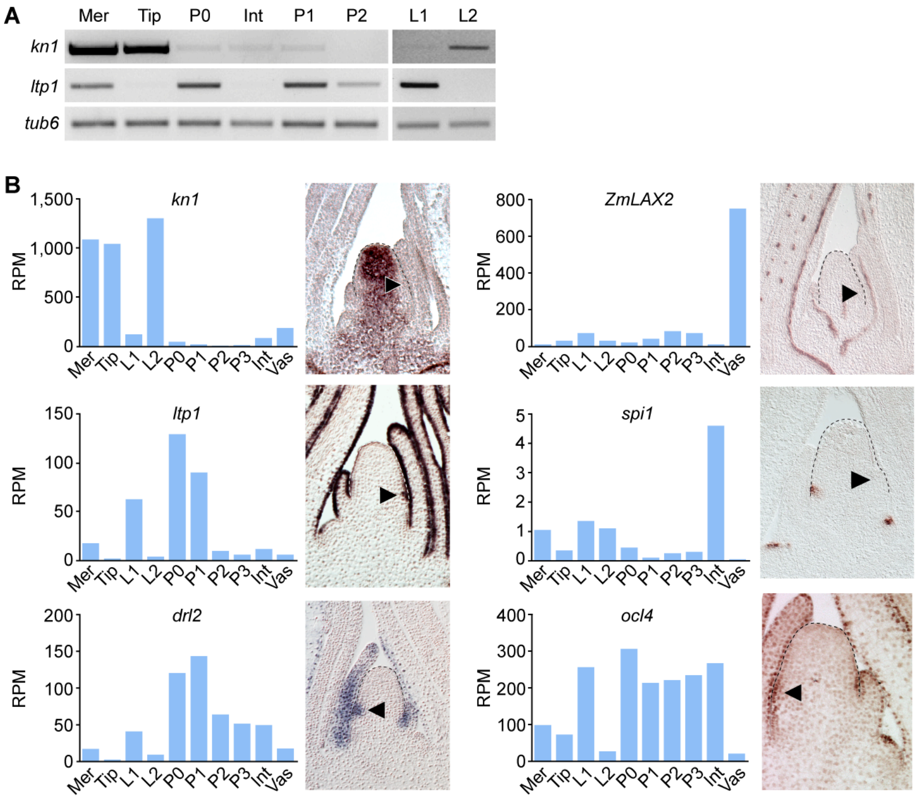

Supplemental Figure 1. Precision of LCM. (*A*) RT-PCR for marker genes *kn1* and *ltp1* on amplified RNA from laser captured domains reveals a high precision of laser microdissection. Consistent with their known expression domains, *kn1* transcripts are present at high levels in the L2 of the meristem and Tip and barely detectable in the L1, internode, and developing leaf primordia, whereas *ltp1* transcripts are detectable specifically in the epidermis of incipient and developing leaf primordia. Mer, meristem; Int, internode. (*B*) Transcript levels for the marker genes *kn1, ltp1, outer cell layer4* (*ocl4*), *drooping leaf ortholog2* (*drl2*), *sparse inflorescence1* (*spi1*), and *like auxin resistant2* (*ZmLAX2*) across the domain RNA-seq libraries reflect their previously reported *in situ* expression patterns, further confirming the precision of microdissections. See **Supplemental Table S15** for gene IDs and references.

Supplemental Figure 2

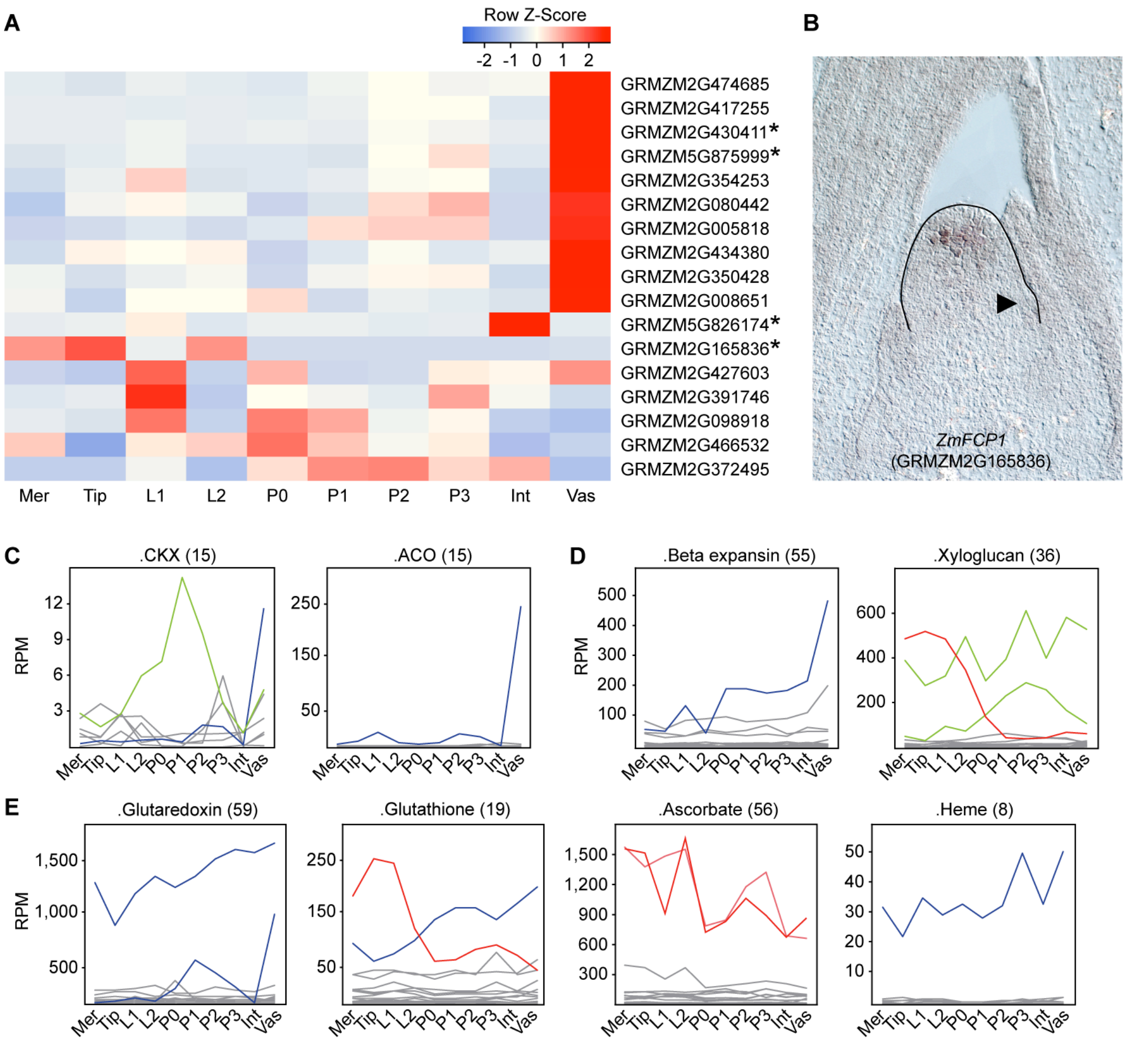

Supplemental Figure 2. Predominant differential expression of individual gene family members distinguishes cell identities. (*A*) Heatmap displaying expression profiles of expressed *CLE* genes. Asterisk, cell type specific *CLEs*. (*B*) *In situ* hybridization shows *FCP1* transcripts accumulate in the L2 of the Tip. Arrowhead, P0. (*C*-*E*) Additional examples of gene families in which individual or select members show abundant and differential expression across the apex. Examples are shown for gene families with functions related to hormone signaling (*C*), cell wall remodeling (*D*), or redox regulation (*E*). Number of members in the family is shown in parenthesis. Only genes expressed at levels ≥ 2 RPM are shown. Mer, meristem; Int, internode; Vas, vasculature.

Supplemental Figure 3

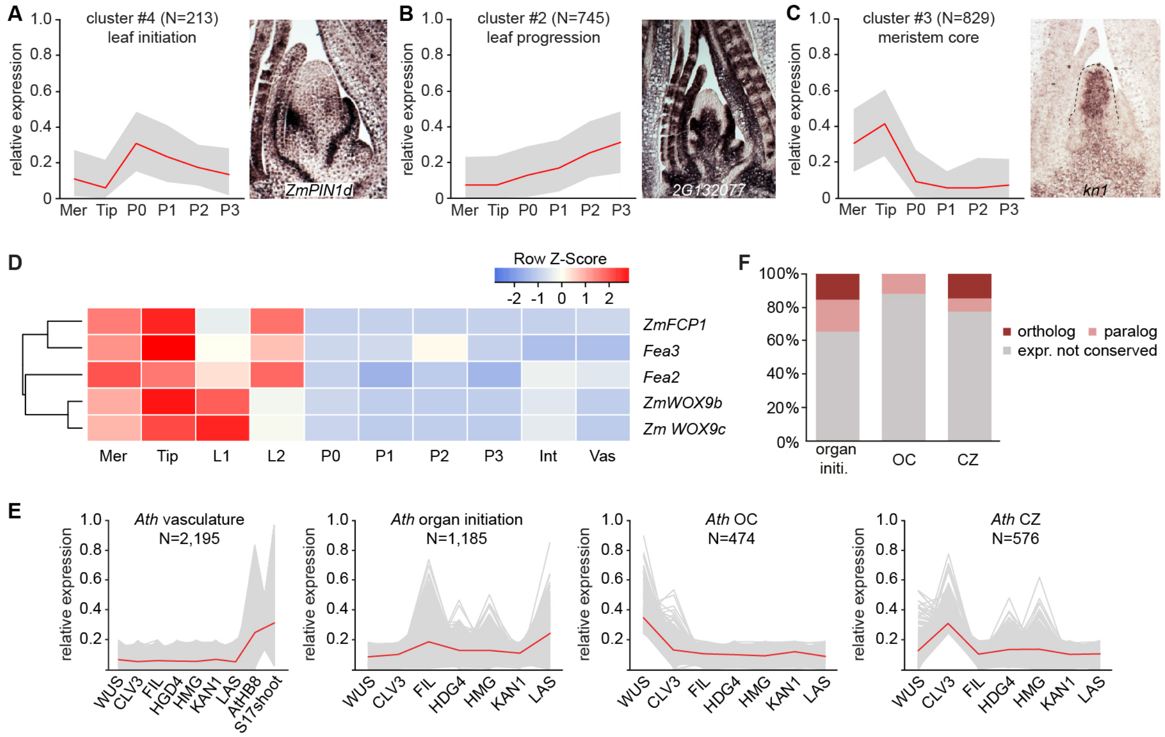

Supplemental Figure 3. Divergent gene sets define functional SAM domains in maize versus *Arabidopsis*. (*A*-*C*) Left: main gene clusters with expression profiles characteristic for leaf initiation (*A*), leaf progression (*B*) or meristem core identity (*C*). Right: representative *in situ* hybridization images. Red line, mean expression values; Grey profile, range between highest and lowest expression values. (*D*) Heatmap displaying expression profiles of key genes in the proposed SAM CLE-WOX signaling module. *ZmFCP1* is expressed specifically in subepidermal layers of the CZ, expression overlaps with that of receptors *FEA2* and *FEA3*. In contrast, expression of *ZmWOX9b* and *ZmWOX9c* specifically marks the L1 of the CZ. Mer, meristem; Int, internode; Vas, vasculature. (*E*) Expression profiles of all domain-enriched *Arabidopsis* genes considered for comparative analyses obtained from Yadav et al. 2009. Red line, mean expression values. Grey lines, individual expression profiles; N, Number of genes. (*F*) Percentages of maize genes expressed specifically during organ initiation, in the OC, or CZ with a similarly expressed *Arabidopsis* ortholog (dark red) or related paralog (light red). Domain enriched *Arabidopsis* genes for this analysis were obtained from Tian et al. 2019. Maize genes without an identifiable *Arabidopsis* ortholog are not shown.

Supplemental Figure 4

A

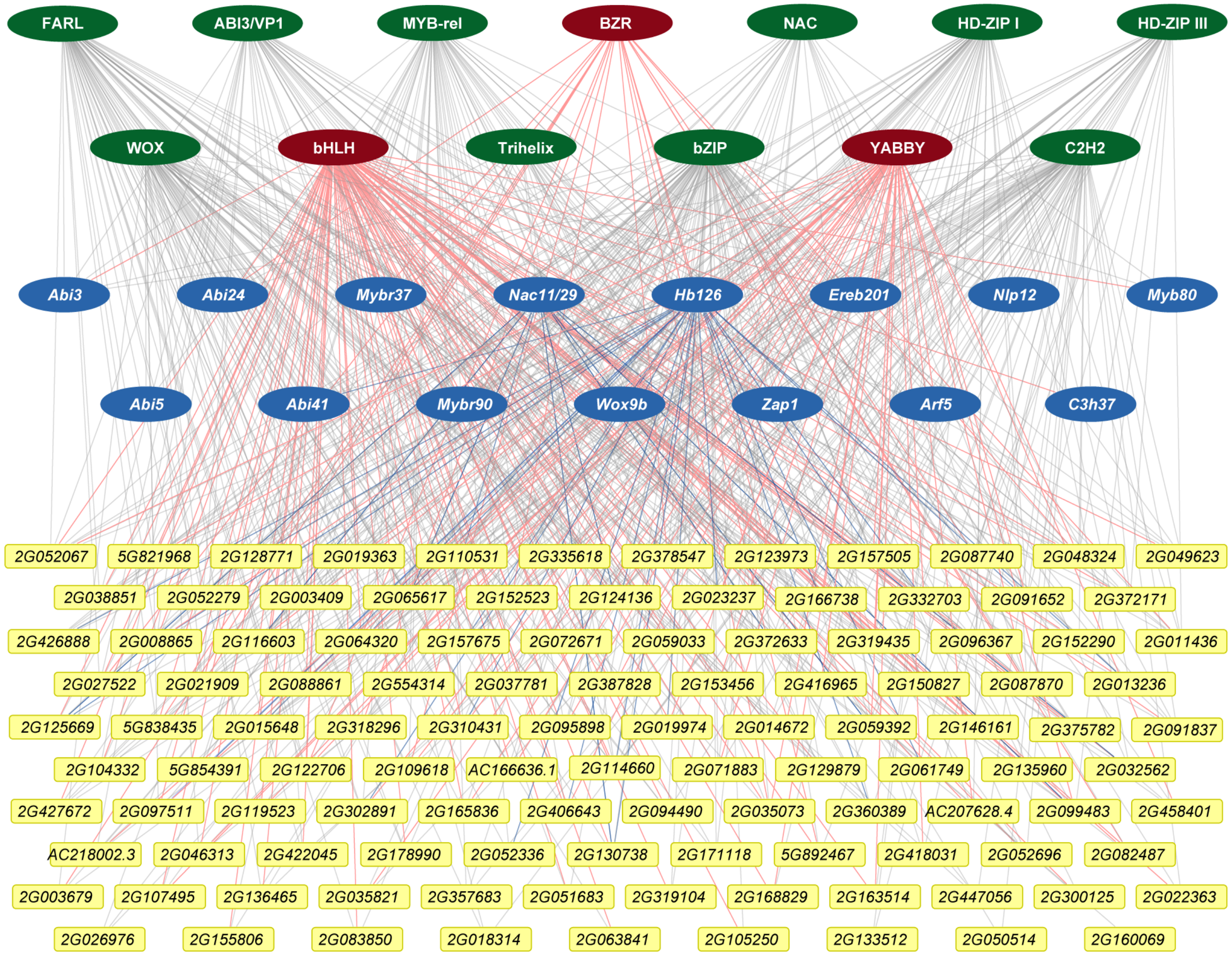

B

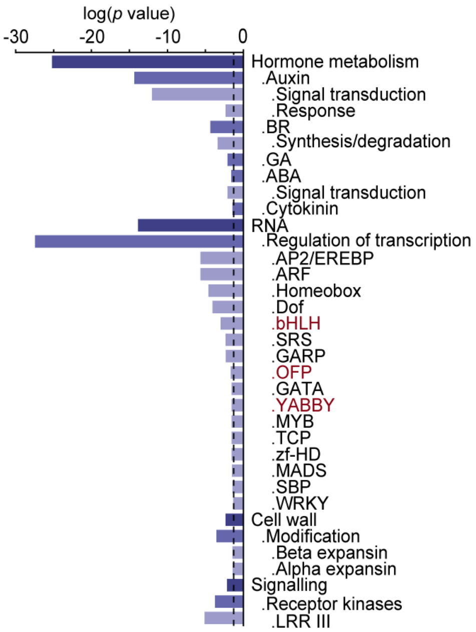

Supplemental Figure 4. Combinatorial effects of diverse TFs distinguishes cell identities. (*A*) Visualization of a GRN for all CZ-specific genes (yellow) with combinatorial interactions from TF families positively- (green) and negatively-correlated (red) to PC1, as well as individual CZ-specific TFs (blue). CZ-specific TFs (blue) can themselves be targeted by more broadly expressed PC1-correlated TF families revealing a hierarchy in the GRN. (*B*) Enrichment analysis on all expressed KN1 targets reveals KN1 preferentially targets genes connected to organogenesis. Color intensity reflects different Mapman category ranks. TF families shown in red are negatively-correlated to PC1 and meristem identity.

Supplemental Figure 5

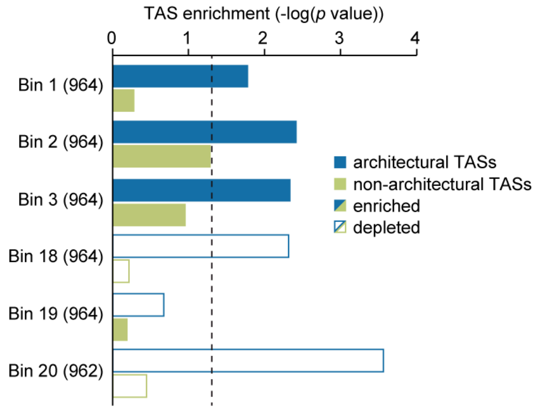

Supplemental Figure 5. Dynamically expressed genes shape morphological variation. Significance plot illustrating that natural variation underlying plant architectural traits is significantly enriched near genes with highly dynamic patterns of expression across the apex. Apex-expressed genes were grouped into 20 bins based on SE score. Architectural TASs are significantly enriched near genes with low SE scores (Bins 1-3), whereas most constitutive expressed genes with high SE scores (Bins 18-20) are depleted for TASs. Dashed line, significance threshold ($p = 0.05$) based on Chi-square test with Yates' continuity correction.
